# Morphological and Mitochondrial Genomic Characterization of Eyeworms (*Thelazia callipaeda*) from Clinical Cases in Central China

**DOI:** 10.3389/fmicb.2017.01335

**Published:** 2017-07-13

**Authors:** Xi Zhang, Ya L. Shi, Zhong Q. Wang, Jiang Y. Duan, Peng Jiang, Ruo D. Liu, Jing Cui

**Affiliations:** Department of Parasitology, School of Basic Medical Sciences, Zhengzhou University Zhengzhou, China

**Keywords:** *Thelazia callipaeda*, thelaziasis, mitochondrial genome, next generation sequencing, phylogeny

## Abstract

*Thelazia callipaeda*, also called the oriental eyeworm, is the major etiological agent of human thelaziasis. Cases of thelaziasis have increased in recent years in China. Although this species is of medical importance, the genetics and phylogenetic systematics of *T. callipaeda* are poorly understood. In this study, we first reported three cases of thelaziasis in central China. All clinical isolates were identified as *T. callipaeda* according to morphological characteristics by light microscopy and scanning electron microscopy. Next, complete mitochondrial (mt) genomes for the three *T*. *callipaeda* isolates from different geographical locations were fully characterized using an Illumina sequencing platform. In addition, all available mt genomes of spirurid nematodes in GenBank were included to reconstruct the phylogeny and to explore the evolutionary histories of the isolates. The genome features of the *T*. *callipaeda* isolates contained 12 PCGs, 22 transfer RNA genes, two ribosomal RNA genes and a major non-coding region. The mtDNA nucleotide sequences of the *T*. *callipaeda* isolates from different hosts and different locations were similar. The n*ad*6 gene showed high sequence variability among all isolates, which is worth considering for future population genetic studies of *T. callipaeda*. Phylogenetic analyses based on maximum parsimony and Bayesian inference methods revealed close relationships among Thelaziidae, Onchocercidae, Setariidae, Gongylonematidae, Physalopteridae, Dracunculidae, and Philometridae. The monophyly of the *T. callipaeda* isolates from different hosts and distinct geographical locations was confirmed. The entire mt genomes of *T*. *callipaeda* presented in this study will serve as a useful dataset for studying the population genetics and phylogenetic relationships of *Thelazia* species.

## Introduction

*Thelazia callipaeda* Railliet and Henry, 1910 (Nematoda: Spirurida: Thelaziidae), also known as the oriental eyeworm, is the major etiological agent of human thelaziasis (Fuentes et al., 2012). *T. callipaeda* infective larvae, which are transmitted by zoophilic insects of the genus *Phortica*, can parasitize the orbital cavity and associated tissues of humans, causing mild to severe signs and symptoms (Otranto et al., 2005a; Shen et al., 2006). Thelaziasis has a worldwide distribution, but most cases occur in eastern and southeastern Asia (Otranto and Dutto, 2008; Nguyen et al., 2012). Human cases are usually associated with poor health and socioeconomic settings, in which heavily affected domestic and wild animals live in close vicinity with humans (Hodžić et al., 2014). China probably has the largest number of cases of thelaziasis in the world with more than 600 cases reported to date (Shen et al., 2006; Wang et al., 2014). In addition, the cases of thelaziasis in China appear to have increased in recent years (Wang et al., 2014).

In clinical diagnostics, it is difficult to differentiate thelaziasis from allergic conjunctivitis when some of the adult or larval stages of *T. callipaeda* are present in the eyes of infected patients (Liu et al., 2013a). Fortunately, the DNA barcoding approach has been useful for *Thelazia* species (Otranto and Traversa, 2004). Several DNA markers have been applied in the taxonomy and genetic studies of *T. callipaeda*, such as cytochrome *c* oxidase subunit 1 gene, *cox1* (Otranto et al., 2005b; Nguyen et al., 2012; Wang et al., 2014; Mihalca et al., 2015), and the first internal transcribed spacer of ribosomal DNA, ITS1 (Otranto and Traversa, 2004). However, in comparison with a single gene, the complete mitochondrial (mt) genome can provide more markers for molecular identification, and provide the potential to discover population variants or cryptic species (Ramesh et al., 2012; Zhang et al., 2016). In recent years, several remarkable advances have been made in the different taxonomic levels of spirurid nematodes using complete mt genomes (Yatawara et al., 2010; Park et al., 2011; McNulty et al., 2012). The first complete mtDNA sequence of *T. callipaeda* has been sequenced and characterized (Liu et al., 2013a). These available mtDNA datasets offer opportunities to compare the diversity of mt genomes among closely related *Thelazia* taxa. In this study, we first describe three cases of thelaziasis in central China. Additionally, the worms collected from patients were sequenced using next generation sequencing (NGS) technology to perform a mitogenomics comparative analysis of spirurid nematodes and to discuss the implications of the new dataset as a new resource for future genetic studies of *T. callipaeda* populations.

## Materials and Methods

### Ethics Statement

This study was approved by the Life Science Ethics Committee of Zhengzhou University (No. 2016-0096). All worms were collected from eyes of children after their legal guardians provided permission. These worm samples were collected not with the purpose of the present study but were removed from patients to treat the thelaziasis.

### Sampling and Data Collection

Three samples of *T. callipaeda* named HeN-ZZ1, HeN-LY1, and HeN-PDS1 were harvested from three patients from different geographical locations of central China (details are shown in the ‘cases report’ part). In addition, all related complete mt genomes available in the GenBank database were included in this study (**Table 1**). To date (January 1, 2017), GenBank contains 24 mitogenomes of spirurid nematodes representing the following eight families: Dracunculidae (1 mtDNAs), Philometridae (1), Onchocercidae (13), Setariidae (1), Gnathostomatidae (3), Physalopteridae (1), Gongylonematidae (1) and Thelaziidae (3). Two *Ascaris* nematodes, *Ascaris lumbricoides* (HQ704900) and *A. suum* (HQ704901), were used as the outgroup.

**Table 1:** The taxa of complete mitochondrial genomes of spirurid nematodes used for this study.

Family	Taxon	Host	Location	Accession number	Reference
Dracunculidae	*Dracunculus medinensis*	N/a	N/a	NC_016019	N/a
Philometridae	*Philometroides sanguineus*	Carassii	China	NC_024931	Su et al., 2016
Onchocercidae	*Acanthocheilonema viteae*	Mongolian jird	N/a	NC_016197	McNulty et al., 2012
	*Brugia malayi*	N/a	N/a	NC_004298	Ghedin et al., 2007
	*Brugia pahangi*	N/a	N/a	AP017680	N/a
	*Brugia timori*	N/a	N/a	AP017686	N/a
	*Chandlerella quiscali*	*Quiscalus quiscula*	United States	NC_014486	McNulty et al., 2012
	*Dirofilaria immitis*	*Canis familiaris*	Australia	NC_005305	N/a
	*Dirofilaria repens*	N/a	N/a	NC_029975	N/a
	*Dirofilaria hongkongensis*	*Homo sapiens*	India	NC_031365	Yilmaz et al., 2016
	*Loa loa*	*Homo sapiens*	Cameroon	NC_016199	McNulty et al., 2012
	*Onchocerca flexuosa*	N/a	N/a	AP017692	N/a
	*Onchocerca ochengi*	N/a	N/a	AP017693	N/a
	*Onchocerca volvulus*	N/a	N/a	AP017695	N/a
	*Wuchereria bancrofti*	N/a	N/a	AP017705	N/a
Setariidae	*Setaria digitata*	N/a	Sri Lanka	GU138699	Yatawara et al., 2010
Gnathostomatidae	*Gnathostoma doloresi*	Wild boar	China	NC_032073	Sun et al., 2016
		Wild boar	Japan	KX231806	Sun et al., 2016
	*Gnathostoma spinigerum*	*Monopterus albus*	China	NC_027726	Liu et al., 2015
Physalopteridae	*Heliconema longissimum*	*Anguilla japonica*	Japan	NC_016127	Park et al., 2011
Gongylonematidae	*Gongylonema pulchrum*	N/a	N/a	AP017685	N/a
Thelaziidae	*Spirocerca lupi*	dog	China	NC_021135	Liu et al., 2013b
	*Thelazia callipaeda*	N/a	N/a	AP017700	N/a
		dog	China	NC_018363	Liu et al., 2013a
		*Homo sapiens*	China	KY908318	This study
		*Homo sapiens*	China	KY908319	This study
		*Homo sapiens*	China	KY908320	This study


### Morphological Identification

The nematodes collected from the patients were transferred to Petri dishes containing physiological saline (0.9% NaCl). The eyeworms were fixed as previously described (Otranto et al., 2004) and examined by light microscopy (Leica Micro system, German) at different magnifications, as well as under the scanning electron microscopy (SEM). All important morphological characteristics of *T. callipaeda* were evaluated for adult forms as reported in Otranto et al. (2003).

### Extraction of Genomic DNA and Library Preparation

Genomic DNA of each specimen was extracted and purified using EasyPure Genomic DNA Kit (Transgen, China) following the manufacturer’s instructions. The complete mt genome was amplified by long-PCR as two overlapping amplicons from the genomic DNA using primers described by Liu et al. (2013a). Next, these two overlapping products were mixed in approximately equal amounts after determining the concentration of each amplicon. The normalized mixed DNA was fragmented to an average size of 500 bp using Covaris M220 system (Covaris, Woburn, MA, United States). The library was prepared using TruSeq DNA PCR-Free Sample Preparation Kit (Illumina, United States) following the manufacturer’s protocols. After these libraries were purified, the DNA concentration of all samples were quantified and 30 ng of each were pooled together. Finally, the oligonucleotide mix was sequenced on an Illumina HiSeq 2000 at the Genewiz Company (Beijing, China).

### Mitogenome Assembly and Annotation

The quality of original sequencing reads was evaluated using the FastQC v.0.11.5 (Andrews, 2010). All ambiguous nucleotides and reads with an average quality value (lower than Q20) were excluded from further analysis. The trimmed sequences were mapped against two reference mt genomes of *T. callipaeda* worms (NC_018363 and AP017700) using the CLC Genomic Workbench v.7.0.4 (Qiagen, Germany). The mapped sequences were later subjected to *de novo* assembly. Contigs with hits to mitochondrial genes or genomes were identified and extracted from the CLC Genomic Workbench. A contig identified as mt genome was manually examined for repeats at the beginning and end of the sequence to establish a circular mtDNA. It was then annotated with MITOS (Bernt et al., 2013) followed by manual validation of the coding regions using the NCBI ORF Finder^1^. The sequin file generated from MITOS was edited and submitted to GenBank with the following accession numbers: KY908318, KY908319, and KY908320.

### Phylogenetic Analysis

Nucleotide sequences of the 12 protein-coding genes (PCGs) were separately aligned using MEGA v.6.06 (Tamura et al., 2013). The sequences of *rrnS*, *rrnL*, and mt-tRNA genes were aligned in MAFFT v.7 (Katoh and Standley, 2013). The program DAMBE v5.2 (Xia and Xie, 2001) was used to measure the nucleotide substitution saturation using the method of Xia et al. (2003) as the substitution saturation masked the phylogenetic signal. Phylograms were constructed using the maximum parsimony (MP) and Bayesian inference (BI) methods, respectively. The MP analyses were performed in PAUP^∗^4b10 (Swofford, 2003) using heuristic searches with TBR branch swapping and 10,000 random addition sequences. Confidence in each node was assessed by boot-strapping (1000 pseudo-replicates, heuristic search of 20 random addition replicates with TBR option). BI analysis was performed in MrBayes v.3.2 (Ronquist et al., 2012) with 10, 000, 000 generations and sampling trees every 100 generations. The best-fit nucleotide substitution model for each data partition was selected by jModelTest 2 (Darriba et al., 2012) under the Akaike Information Criterion (AIC). Stationarity was assessed using a convergence diagnostic. An average standard deviation of the split frequencies (ASDSF) < 0.01 was used as the criteria of convergence between both runs. The consensus tree was drawn after removing the first 20 000 trees (20%) as the burn-in phase. In addition, the data used in the phylogenetic analyses were partitioned under three schemes: partitioned among PCGs (12 partitions, *nad*5, *cox*3, *cytb*, *nad*4L, *nad*4, *atp*6, *nad*2, *nad*1, *nad*3, *cox*1, *cox*2, and *nad*6), partitioned by rRNA genes (2 partitions, *rrnL* and *rrnS*), and partitioned by PCGs and rRNA (14 partitions).

## Results

### Cases Descriptions and Parasite Identification

The first case was a 6-year-old boy from Lushan county of Pingdingshan city, Henan province (33°36′ N, 112°25′ E) in central China. In September 2014, the boy was admitted to the First Affiliated Hospital of Zhengzhou University with a 7-day history of creeping sensation, itching, and increased secretions in his right eye, but without any systemic or visual symptoms. His medical history was notable only for an unknown insect flying into his right eye approximately 2 months earlier. On ophthalmological examination, several thread-like worms were seen moving in the nasal upper eyelid of the eyes above the conjunctiva. The worms nested in the subconjunctiva tissue were completely cleared with intraocular forceps. Four worms were removed, 1 in the left eye and 3 in the right eye. The second case was a 2.5-year-old boy who lived in the suburbs of Zhengzhou city, Henan province (34°40′ N, 113°12′ E). He visited his doctor in August 2015 with an approximately 2 weeks history of itching, conjunctival hyperemia and increased secretions in eyes. Ophthalmological examination found six slender worms on the conjunctival sac of the left eye. No additional abnormal manifestations were noted on physical examination. The symptoms disappeared after the worms were removed with forceps. The third case was a 1.5-year-old child from a village in Luoyang, Henan province (34°43′ N, 112°42′ E). The clinical manifestation was similar to the second case, and five worms were removed in her conjunctival sac, four in the left eye and one in the right eye.

**FIGURE 1:**
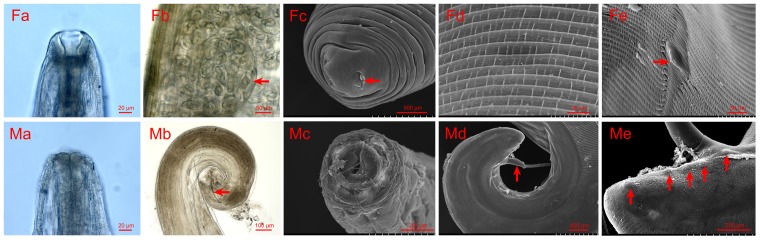
Photomicrographs and scanning electron micrographs (SEMs) of female and male *Thelazia callipaeda*. **(Fa)** Light micrograph of female anterior head and buccal capsule. **(Fb)** Light micrograph of immature eggs or germ cells filling the uterine tubules of female. **(Fc)** SEM of the tip of the female tail and the femal anus. **(Fd)** SEM of the transversally striated cuticle on the anterior of female. **(Fe)** SEM of the anterior region of female, vulva with a short vulvar flap. **(Ma)** Light micrograph of male anterior head. **(Mb)** Light micrograph of the male caudal end and detail of the shorter right spicule. **(Mc)** SEM of anterior region of the male. **(Md)** SEM of caudal end of male. **(Me)** SEM of caudal end of male, five pairs of postcloacal papillae.

All worms collected from the patients were identified as *T. callipaeda* according to the morphological characteristics described by Otranto et al. (2003). More specifically, for the females, the following characteristics were noted: (1) *T. callipaeda* was characterized by the presence of a buccal capsule (**Figure 1Fa**); (2) in the posterior half of the body, immature eggs or germ cells filled the uterine tubules (**Figure 1Fb**); (3) an anus was present on the tip of the tail (**Figure 1Fc**); (4) the transversally striated cuticle was more corrugated on the anterior of the body than on the caudal end (**Figure 1Fd**); and (5) the vulva with a short vulvar flap was located in the anterior region of the body (**Figure 1Fe**). For the males, the following characteristics were noted: (1) in contrast with the female, the buccal capsule was inconspicuous (**Figure 1Ma**), and the mouth opening showed a cycle or ellipse profile (**Figure 1Mc**); (2) two dissimilar spicules were present in the males, one was the anterior extremity of the longer left spicule and other was the typical crescent shape of the shorter right spicule (**Figure 1Mb**); (3) the caudal end was ventrally curved (**Figure 1Md**); and (4) there were five pairs of postcloacal papillae (**Figure 1Me**).

### Assembly of the mt Genome

**FIGURE 2:**
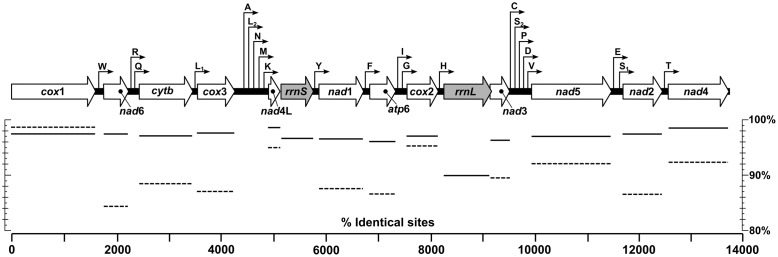
Linear maps of the mitochondrial genomes of *T. callipaeda*. Outline arrows indicate the positions and direction of transcription of protein- and rRNA-coding genes, hairline arrows indicate the position of tRNA encoding genes (*trn*). Solid and dotted horizontal lines below depict average nucleotide and amino acid identities of aligned protein- and rRNA-coding regions of specimens from Luoyang (HeN-LY1), Pingdingshan (HeN-PDS1) and Zhengzhou (HeN-ZZ1) of central China and Guangdong (NC_018363) and Japan (AP017700), respectively. *atp*6, adenosine triphosphatase subunit 6; *cox*, cytochrome *c* oxidase complex; *cytb*, cytochrome *b*; *nad*, nicotinamide dehydrogenase complex; *rrnL*, large subunit rDNA; *rrnS*, small subunit rDNA.

A total of 961 424, 961 094, and 970 104 sequence reads with a total of 162 140 093, 166 174 524, and 162 301 339 bases were mapped to full mitochondrial genomes for the *T. callipaeda* isolates of HeN-LY1, HeN-PDS1, and HeN-ZZ1, respectively (Supplementary Table S1). All three novel mt genomes were fully annotated and each encoded an identical set of 12 PCGs, 22 tRNA genes, and 2 rRNA genes. All genes were encoded in an identical order and direction, and all PCGs annotated across the three representatives of *T. callipaeda* were of the same length (**Figure 2**). Several genes overlapped at the boundaries, such as *nad*6/*trn*R (9 bp), *trn*L_1_/*cox*3 (3 bp), *trn*L_2_/*trn*N (11 bp), *nad*1/*trn*F (32 bp), *cox*2/*trn*H (10 bp), *rrnL*/*nad*3 (8 bp), *trn*S_1_/*nad*2 (3 bp), and *trn*T/*nad*4 (6 bp) (Supplementary Table S2). All PCGs had ATG, TTT, ATA, and TTG as the initiation codons and TAA or TAG as the termination codons. However, the TAT termination codon was used only in the *nad1* gene. The ribosomal RNA genes *rrnL* and *rrnS* were 965 and 666 bp long, respectively. The *rrnL* was located between *trn*H and *nad*3, and *rrnS* was between *nad*4L and *trn*Y. The lengths of the 22 tRNA genes ranged from 53 to 66 nucleotides (nt). A major non-coding region located between *cox*3 and *trn*A was interspersed in *T. callipaeda* isolates.

The mtDNA nucleotide sequences from the *T. callipaeda* isolates from different locations were conspicuously similar. The full-length mt genomes were identical in 98.22% nucleotides of the 13 668 bp long alignment of the three isolates. When published *T. callipaeda* mt sequences from Guangdong of China (isolated from dog, No. NC_018363) and Japan (No. AP017700) were added, the similarities of the mt genomes declined to 96.89%. Pairwise genetic distances between individual pairs of these isolates measured as a percentage of identical nucleotides ranged from 98.06% (Guangdong and HeN-LY1 isolates) to 99.53% (HeN-ZZ1 and Japan isolates). Comparing the protein-coding gene regions (**Figure 2**), *T. callipaeda* displayed high levels of sequence conservation ranging from 96.05% of identical nucleotides in *atp*6 to 98.74% in *nad*4L. The amino acid sequence similarities ranged from 84.31% (*nad*6) to 98.73% (*cox*1). The most divergent amino acid sequence was *nad*6 between the HeN-PDS1 and Japan isolates. The mt large and small subunit rDNAs (*rrnL*, *rrnS*) shared 89.95 and 96.71% identical nucleotides, respectively.

### Phylogeny Reconstruction

**FIGURE 3:**
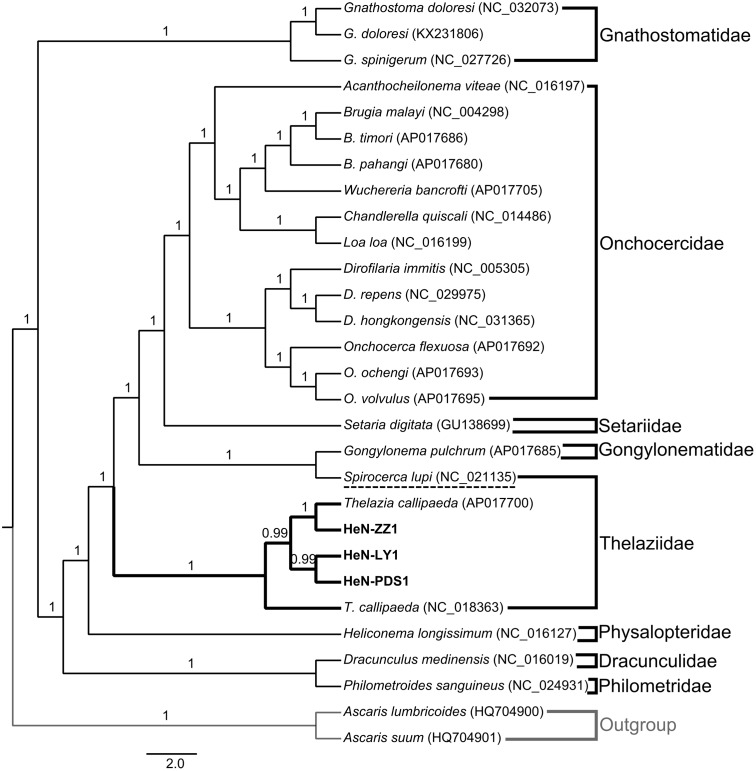
Bayesian phylogenetic tree of collected spirurid nematodes based on the analysis of 12 protein-coding genes (PCGs) and 2 rRNA genes. Numbers above branches represent the Bayesian posterior probabilities. Only posterior probabilities above 0.6 are shown.

The test of substitution saturation showed that the observed indexes of substitution saturation (*Iss*) for all alignments were significantly lower than the corresponding critical index substitution saturation (*Iss.c*), indicating that there was little saturation in the present sequences (**Table 2**). The likelihood models identified by the jModelTest (AIC) suggested that the GTR model was most suitable for included data partitions (Supplementary Table S3). A total of six analyses by two phylogenetic inference methods (MP and BI) under three partition schemes (partitioned among PCGs, partitioned by rRNA genes, and PCGs + rRNA) were completed in this study. Generally, the trees generated from different methods with different partition schemes shared similar topologies (**Figure 3** and Supplementary Figures S1–S5). Among spirurid nematodes, the family Gnathostomatidae was a sister group to the remaining families: Dracunculidae, Philometridae, Physalopteridae, Thelaziidae, Gongylonematidae, Setariidae, and Onchocercidae (**Figure 3**). The earliest diversifications among these remaining families gave rise to Dracunculidae and Philometridae and then to Physalopteridae. In addition, Dracunculidae and Philometridae composed clade with high support value (posterior probabilities = 1.0). The next diversification event would have separated Thelaziidae, Gongylonematidae, Setariidae, and Onchocercidae, in which the Thelaziidae and Gongylonematidae were basal. The Onchocercidae as a monophyletic group (pp = 1.0) was the last diverged. However, the monophyly of family Thelaziidae was unresolved: the *T. callipaeda* isolates from different geographical locations generated a single group (pp = 1.0), and the other species *Spirocerca lupi* plus Gongylonematidae made up a separate clade.

**Table 2:** Results of conserved sites (Cs), variable sites (Vs), parsim-info sites (Ps), singleton sites (Ss) and test of substitution saturation of aligned sequences of spirurid nematodes.

Partition	Final length	Cs	Vs	Ps	Ss	*Iss^∗^*	*Iss.c* #(*P*-value)
*atp*6	603	68	535	453	78	0.5074	0.6998 (0.0000)
*cox*1	1656	645	1011	845	166	0.3036	0.7820 (0.0000)
*cox*2	711	183	528	442	83	0.3929	0.7396 (0.0000)
*cox*3	783	193	590	516	74	0.4288	0.7478 (0.0000)
*cytb*	1095	381	714	596	118	0.3527	0.7652 (0.0000)
*nad*1	909	284	625	495	130	0.3838	0.7537 (0.0000)
*nad*2	867	142	725	564	161	0.4853	0.7451 (0.0000)
*nad*3	345	79	266	225	41	0.5216	0.6615 (0.0000)
*nad*4	1233	352	881	700	181	0.3961	0.7721 (0.0000)
*nad*4L	243	26	217	179	38	0.5893	0.6048 (0.0000)
*nad*5	1599	397	1202	979	223	0.4461	0.7615 (0.0000)
*nad*6	471	51	420	336	84	0.5625	0.6901 (0.0000)
*rrnL*	1003	233	770	592	178	0.3559	0.7514 (0.0000)
*rrnS*	686	202	484	393	91	0.3135	0.6901 (0.0000)


*^∗^Iss indicates index of substitution saturation. #Iss.c denotes critical index substitution saturation for an asymmetrical tree*.

## Discussion

Thelaziasis can occur when infected flies of the genus *Phortica* feed on the ocular secretions, tears, and conjunctiva of hosts (Fuentes et al., 2012). In China, since the first case with human thelaziasis was described in 1917, more than 600 cases scattered across almost the entire country have been reported (Wang et al., 2014). Human cases typically occur in rural villages associated with poor health and socioeconomic settings (Shen et al., 2006). Until now, the etiological agent of all human thelaziasis reported in China was identified as *T. callipaeda*, but few cases reported detailed morphological characteristics. In this study, we firstly reported three cases with clinical eyeworms in central China, and we examined these worms under light microscopy and scanning electron microscopy. The key characteristics of the *T. callipaeda* isolates in our study were similar to those of *T. callipaeda* isolates from dogs in southern Italy (Otranto et al., 2003). In addition, the mouth profile of the *T. callipaeda* male was first described in this study. In comparison with the isolates from Italy, the sizes of the parasites collected here were relatively smaller; the males measured 6.4–10.3 mm in length and 307–389 μm in width (7.7–12.8 mm in length and 338–428 μm in width for the Italy isolates), and the females measured 11.2–15.3 mm in length and 311–459 μm in width (12–18.5 mm in length and 370–510 μm for the Italy isolates).

In recent years, the mitochondrial genomes of spirurid nematodes have been extensively studied (Ghedin et al., 2007; Yatawara et al., 2010; Park et al., 2011; McNulty et al., 2012; Liu et al., 2015; Yilmaz et al., 2016). These whole mt genomes will serve as a useful dataset for studying the genetics, systematics and phylogenetic relationships of spirurid nematodes. In accordance with the two known mt genomes of *T. callipaeda* (NC_018363 and AP017700), all *T. callipaeda* isolates from central China contained 12 PCGs, 22 transfer RNA genes, two ribosomal RNA genes and a non-coding region but lacked an *atp*8 gene (Liu et al., 2013a). The gene content and arrangement are the same as those of *Acanthocheilonema viteae*, *Brugia* species, *Chandlerella quiscali*, *Dirofilaria* species, *Loa loa*, *Onchocerca* species, *Wuchereria bancrofti*, *Gongylonema pulchrum*, *Setaria digitata* (Ghedin et al., 2007; Yatawara et al., 2010; McNulty et al., 2012; Yilmaz et al., 2016), but distinct from those of *Dracunculus medinensis*, *Philometroides sanguineus*, *Gnathostoma doloresi*, and *Heliconema longissimum* (Su et al., 2016; Sun et al., 2016). The mt genome sequence of *T. callipaeda* has high a A+T content (above 70%), which is consistent with the mt genomes of nematodes from the order Spirurida (Yatawara et al., 2010; McNulty et al., 2012). All PCGs had ATG, TTT, ATA, or TTG as their initiation codons, and TAA or TAG as their termination codons, which was consistent with a previous study (Liu et al., 2013a). The sizes of the 22 tRNA genes identified here were similar to those of other spirurid nematodes, indicating their similar functions (Liu et al., 2013b; Yilmaz et al., 2016). The locations of *rrnL* and *rrnS* were also identical to previously published mt genomes of *Thelazia*. The nucleotide sequences of the mtDNA of five *T. callipaeda* isolated from different geographical locations were conspicuously similar, which indicated that the influence of genetic variations on the phylogenetic distances in *T. callipaeda* is relatively small (Zhang et al., 2015). The mt genome of the *T. callipaeda* isolate from Guangdong (NC_018363) was the most divergent of the entire set. The results of sequence divergence of PCGs suggested that *nad*4L is the most conserved protein-coding gene. *Nad*6 showed high sequence variability among the five isolates, indicating that this gene is suitable as a genetic marker for population genetic studies of *T. callipaeda* from different geographic origins.

Although nuclear ribosomal genes (rDNA) have been verified for phylogenetic studies of a wide range of organisms (Nadler et al., 2007; Zhang et al., 2017), in comparison with a single gene or a single region of rDNA, the complete mt genome sequences have significantly improved the power of phylogenetic analysis and made possible more accurate resolution of taxonomic relationships even at deep levels (Brabec et al., 2016). For spirurid nematodes, the mt genomes have been applied particularly to studies regarding phylogeny and evolution (Park et al., 2011; Liu et al., 2013a, 2015; Yilmaz et al., 2016). To date, there are 24 mitogenomes of the order Spirurida in GenBank representing eight families. Our study brings a new perspective by presenting additional mt genomic data of *T. callipaeda* from three clinical isolates. These mtDNA sequences will be useful for current molecular taxonomy studies focused on *Thelazia* species. In our phylogenetic analyses, the taxonomic relationships of Spirurida based on different data partitions and different inference methods were generally congruent. In general, the tree topology of selected spirurid nematodes can be divided into two main clades: one small clade only contained the family Gnathostomatidae, and the other large one included Onchocercidae, Setariidae, Gnathostomatidae, Thelaziidae, Physalopteridae, Dracunculidae, and Philometridae. The position of Gnathostomatidae was also supported by Liu et al. (2015). Within the large clade, Dracunculidae, Philometridae, and Physalopteridae were in the basal, which was consistent with previous publications (Park et al., 2011; Liu et al., 2013a, 2015). The close relationships among Onchocercidae, Setariidae, and Gnathostomatidae was verified in studies of Park et al. (2011); McNulty et al. (2012), and Liu et al. (2015). In this study, these relationships have also been supported via the addition of more representative sequences. For Thelaziidae, the monophyly of *T. callipaeda* isolates from different geographical locations was undoubted. However, the position of *Spirocerca lupi* was ambiguous. In the previous studies by Liu et al. (2013a, 2015) and Yilmaz et al. (2016), *S. lupi* was placed in the family Spiruridae. However, in the NCBI taxonomy browser^2^, *Spirocerca lupi* is included within the family Thelaziidae. Increased sampling and molecular marker analysis should be conducted to determine the exact phylogenetic position of *S. lupi*. Our results also supported *Spirocerca lupi* as a single clade with a close relationship to the family Gongylonematidae.

## Conclusion

In this study, three cases of thelaziasis were reported in central China, and all clinical worms were identified as *T. callipaeda* according to several key characteristics examined under light microscopy and scanning electron microscopy. In addition, the whole mitochondrial genomes of three *T. callipaeda* isolates from different geographical locations were sequenced using next-generation sequencing methods. The genome featured 12 PCGs, 22 transfer RNA genes, two ribosomal RNA genes and a non-coding region, which was similar to that of most of other spirurid nematodes. The mtDNA nucleotide sequences of these isolates from different locations were conspicuously similar. The n*ad*6 gene showed high sequence variability among all isolates, which is worth considering for future population genetic studies of *T. callipaeda*. Phylogenetic analyses support the monophyly of *T. callipaeda* isolates from different hosts and distinct geographical locations. However, the taxonomic position of *Spirocerca lupi* is still ambiguous.

## Author Contributions

Conceived and designed the experiments: XZ, ZW, and JC. Performed the experiments: XZ, YS, JD, PJ, and RL. Analyzed the data: XZ. Contributed reagents/materials/analysis tools: JC, XZ, and ZW. Wrote the paper: XZ, ZW, and JC. All authors read and approved the final version of the manuscript.

## Conflict of Interest Statement

The authors declare that the research was conducted in the absence of any commercial or financial relationships that could be construed as a potential conflict of interest.

## Footnotes

1 http://www.ncbi.nlm.nih.gov/gorf/gorf.html

2 https://www.ncbi.nlm.nih.gov/taxonomy
