# Supplementary material for: Morphological and Mitochondrial Genomic Characterization of Eyeworms (Thelazia callipaeda) from Clinical Cases in Central China
Source: Front Microbiol. 2017 Jul 13;8:1335. doi: 10.3389/fmicb.2017.01335 (PMC5508006; doi:10.3389/fmicb.2017.01335)
Supplement: Supplementary file 1 [file Data_Sheet_1.doc]

**Morphological and mitochondrial genomic characterization of eyeworms (*Thelazia callipaeda*) from clinical cases in central China**

Xi Zhang, Ya Li Shi, Zhong Quan Wang*, Jiang Yang Duan, Peng Jiang, Ruo Dan Liu, Jing Cui*

**Supporting information**

**Table S1.** Sequence statistics of the Illumina sequencing assembly.

**Table S2.** Characteristics of mitochondrial genomes of *Thelazia callipaeda* isolates from central China.

**Table S3.** Best fitting models determined by the Akaike information criterion (AIC) for the data partitions used in this study.

**Figure S1.** Maximum parsimony (MP) phylogenetic tree of collecting spirurid nematodes based on the analysis of 12 protein-coding genes (PCGs) and 2 rRNA genes.

**Figure S2.** Maximum parsimony (MP) phylogenetic tree of collecting spirurid nematodes based on the analysis of PCGs.

**Figure S3.** Maximum parsimony (MP) phylogenetic tree of collecting spirurid nematodes based on the analysis of 2 rRNA genes.

**Figure S4.** Bayesian phylogenetic tree of collecting spirurid nematodes based on the PCG analysis.

**Figure S5.** Bayesian phylogenetic tree of collecting spirurid nematodes based on the analysis of 2 rRNA genes.

**Table S1.** Sequence statistics of the Illumina sequencing assembly.

| Sample | Length of  Mt (bp) | Reads | Length^*^  (bp) | Bases | Q20(%) | GC(%) | N^#^  (ppm) |
| --- | --- | --- | --- | --- | --- | --- | --- |
| HeN-LY1 | 13668 | 961424 | 168.65 | 162140093 | 97.41 | 26.41 | 19.03 |
| HeN-PDS1 | 13667 | 961094 | 172.90 | 166174524 | 97.63 | 26.97 | 16.01 |
| HeN-ZZ1 | 13668 | 970104 | 168.72 | 162301339 | 97.69 | 25.36 | 18.59 |

* The average length of reads; # Per base N content per million bases. Q20 indicate an error probability of 1%.

**Table S2.** Characteristics of mitochondrial genomes of *Thelazia callipaeda* isolates from central China.

| Genes | Length of genes and sequences | | | | | | Codon used for | | | Position in genome (5'-3') | | |
| --- | --- | --- | --- | --- | --- | --- | --- | --- | --- | --- | --- | --- |
|  | Nucleotide | | | Amino acid | | | Initiation/Termination | | |  |  |  |
|  | LY1 | PDS1 | ZZ1 | LY1 | PDS1 | ZZ1 | LY1 | PDS1 | ZZ1 | LY1 | PDS1 | ZZ1 |
| *cox1* | 1653 | 1653 | 1653 | 550 | 550 | 550 | ATG/TAA | ATG/TAA | ATG/TAA | 1-1653 | 1-1653 | 1-1653 |
| *trnW* | 55 | 55 | 55 |  |  |  |  |  |  | 1662-1716 | 1662-1716 | 1662-1716 |
| *nad*6 | 462 | 462 | 462 | 153 | 153 | 153 | TTT/TAA | TTT/TAA | TTT/TAA | 1749-2210 | 1749-2210 | 1749-2210 |
| *trnR* | 66 | 66 | 66 |  |  |  |  |  |  | 2202-2267 | 2202-2267 | 2202-2267 |
| *trnQ* | 53 | 53 | 53 |  |  |  |  |  |  | 2267-2319 | 2267-2319 | 2267-2319 |
| *cytb* | 1086 | 1086 | 1086 | 361 | 361 | 361 | TTT/TAA | TTT/TAA | TTT/TAA | 2324-3409 | 2324-3409 | 2324-3409 |
| *trnL*_1_ | 56 | 56 | 56 |  |  |  |  |  |  | 3409-3464 | 3409-3464 | 3409-3464 |
| *cox*3 | 783 | 783 | 783 | 260 | 260 | 260 | ATA/TAG | ATA/TAG | ATA/TAA | 3462-4244 | 3462-4244 | 3462-4244 |
| *Non-coding region* | 316 | 316 | 316 |  |  |  |  |  |  | 4245-4560 | 4245-4560 | 4245-4560 |
| *trnA* | 60 | 59 | 60 |  |  |  |  |  |  | 4561-4620 | 4561-4619 | 4561-4620 |
| *trnL*_2_ | 60 | 60 | 60 |  |  |  |  |  |  | 4622-4681 | 4621-4680 | 4622-4681 |
| *trnN* | 54 | 54 | 54 |  |  |  |  |  |  | 4671-4734 | 4670-4733 | 4671-4734 |
| *trnM* | 59 | 59 | 59 |  |  |  |  |  |  | 4736-4794 | 4735-4793 | 4736-4794 |
| *trnK* | 58 | 58 | 58 |  |  |  |  |  |  | 4795-4852 | 4794-4851 | 4795-4852 |
| *nad*4L | 237 | 237 | 237 | 78 | 78 | 78 | TTT/TAA | TTT/TAA | TTT/TAA | 4855-5091 | 4854-5090 | 4855-5091 |
| *rrnS* | 666 | 666 | 666 |  |  |  |  |  |  | 5092-5757 | 5091-5756 | 5092-5757 |
| *trnY* | 57 | 57 | 57 |  |  |  |  |  |  | 5757-5813 | 5756-5812 | 5757-5813 |
| *nad*1 | 906 | 906 | 906 | 301 | 301 | 301 | TTG/TAT | TTG/TAT | TTG/TAT | 5814-6719 | 5813-6718 | 5814-6719 |
| *trnF* | 61 | 61 | 61 |  |  |  |  |  |  | 6688-6748 | 6687-6747 | 6688-6748 |
| *atp6* | 582 | 582 | 582 | 193 | 193 | 193 | ATT/TAG | ATT/TAG | ATT/TAG | 6749-7330 | 6748-7329 | 6749-7330 |
| *trnI* | 57 | 57 | 57 |  |  |  |  |  |  | 7332-7388 | 7331-7387 | 7332-7388 |
| *trnG* | 57 | 57 | 57 |  |  |  |  |  |  | 7390-7446 | 7389-7445 | 7390-7446 |
| *cox*2 | 705 | 705 | 705 | 234 | 234 | 234 | ATA/TAG | ATA/TAG | ATA/TAG | 7450-8154 | 7449-8153 | 7450-8154 |
| *trnH* | 57 | 57 | 57 |  |  |  |  |  |  | 8145-8201 | 8144-8200 | 8145-8201 |
| *rrnL* | 965 | 965 | 965 |  |  |  |  |  |  | 8201-9165 | 8200-9164 | 8201-9165 |
| *nad*3 | 336 | 336 | 336 | 111 | 111 | 111 | TTG/TAG | TTG/TAG | TTG/TAG | 9158-9493 | 9157-9492 | 9158-9493 |
| *trnC* | 56 | 56 | 56 |  |  |  |  |  |  | 9494-9549 | 9493-9548 | 9494-9549 |
| *trnS*_2_ | 52 | 52 | 52 |  |  |  |  |  |  | 9549-9600 | 9548-9599 | 9549-9600 |
| *trnP* | 55 | 55 | 55 |  |  |  |  |  |  | 9602-9656 | 9601-9655 | 9602-9656 |
| *trnD* | 56 | 56 | 56 |  |  |  |  |  |  | 9718-9773 | 9717-9772 | 9718-9773 |
| *trnV* | 58 | 58 | 58 |  |  |  |  |  |  | 9774-9831 | 9773-9830 | 9774-9831 |
| *nad*5 | 1593 | 1593 | 1593 | 530 | 530 | 530 | TTG/TAG | TTG/TAG | TTG/TAG | 9830-11422 | 9829-11421 | 9830-11422 |
| *trnE* | 56 | 56 | 56 |  |  |  |  |  |  | 11425-11480 | 11424-11479 | 11425-11480 |
| *trnS*_1_ | 53 | 53 | 53 |  |  |  |  |  |  | 11480-11532 | 11479-11531 | 11480-11532 |
| *nad*2 | 861 | 861 | 861 | 286 | 286 | 286 | TTG/TAA | TTG/TAA | TTG/TAA | 11513-12373 | 11512-12372 | 11513-12373 |
| *trnT* | 58 | 58 | 58 |  |  |  |  |  |  | 12381-12438 | 12380-12437 | 12381-12438 |
| *nad*4 | 1233 | 1233 | 1233 | 410 | 410 | 410 | TTG/TAG | TTG/TAG | TTG/TAG | 12433-13665 | 12432-13664 | 12433-13665 |

**Table S3.** Best fitting models determined by the Akaike information criterion (AIC) for the data partitions used in this study.

| Partition | Model (AIC) | *P*-Inv. | G-shape |
| --- | --- | --- | --- |
| *atp*6 | TN93+G | n/a | 0.58 |
| *cox*1 | GTR+G+I | 0.19 | 0.60 |
| *cox*2 | GTR+G | n/a | 0.49 |
| *cox*3 | GTR+G | n/a | 0.45 |
| *cytb* | TN93+G+I | 0.18 | 0.62 |
| *nad*1 | GTR+G+I | 0.16 | 0.80 |
| *nad*2 | TN93+G | n/a | 0.69 |
| *nad*3 | GTR+G | n/a | 0.70 |
| *nad*4 | GTR+G | n/a | 0.48 |
| *nad*4L | TN93+G | n/a | 0.80 |
| *nad*5 | GTR+G+I | 0.15 | 0.90 |
| *nad*6 | GTR+G | n/a | 0.81 |
| *rrnL* | GTR+G | n/a | 0.58 |
| *rrnS* | TN93+G | n/a | 0.45 |

**
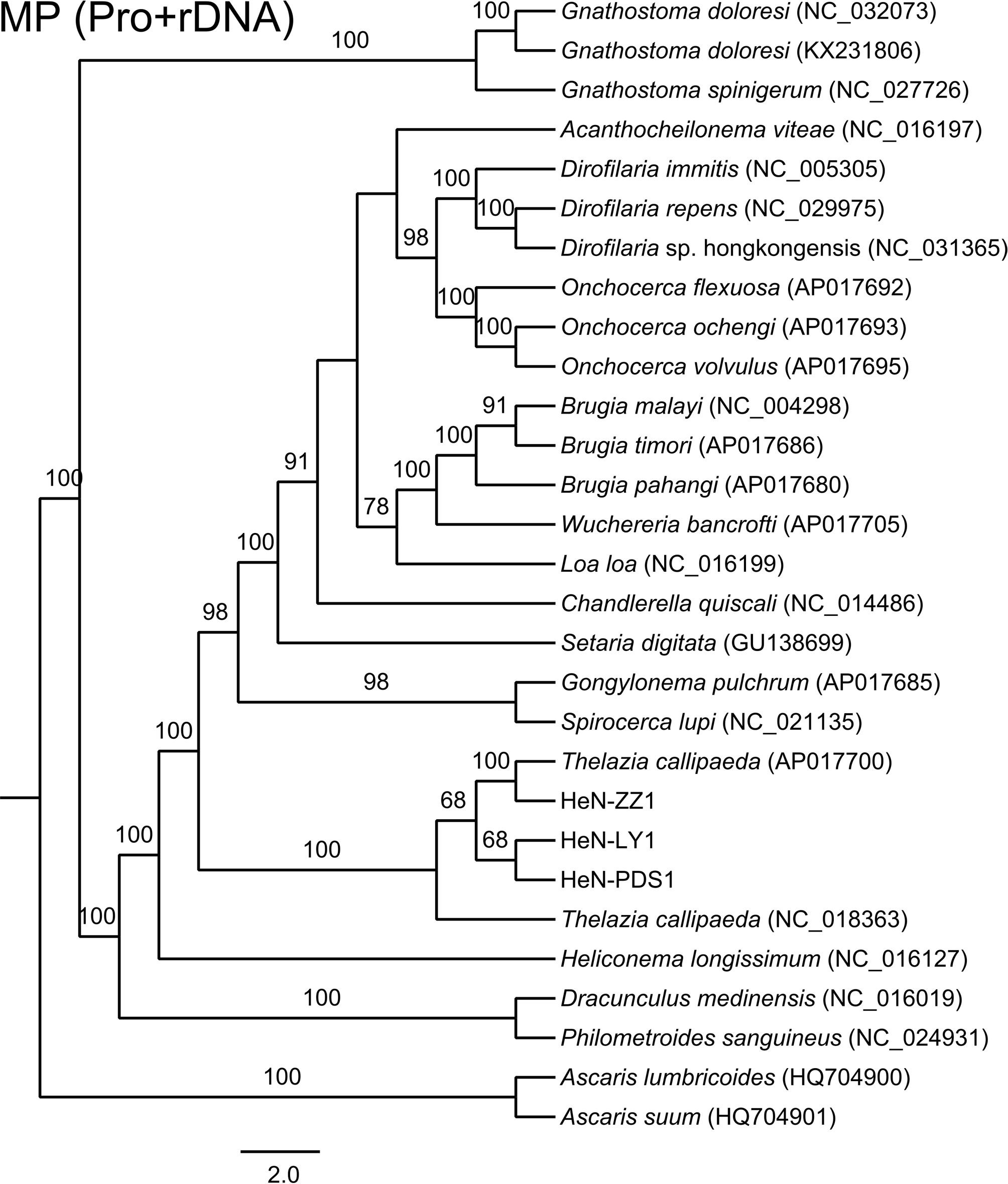
**

**Figure S1.** Maximum parsimony (MP) phylogenetic tree of collecting spirurid nematodes based on the analysis of 12 protein-coding genes (PCGs) and 2 rRNA genes.


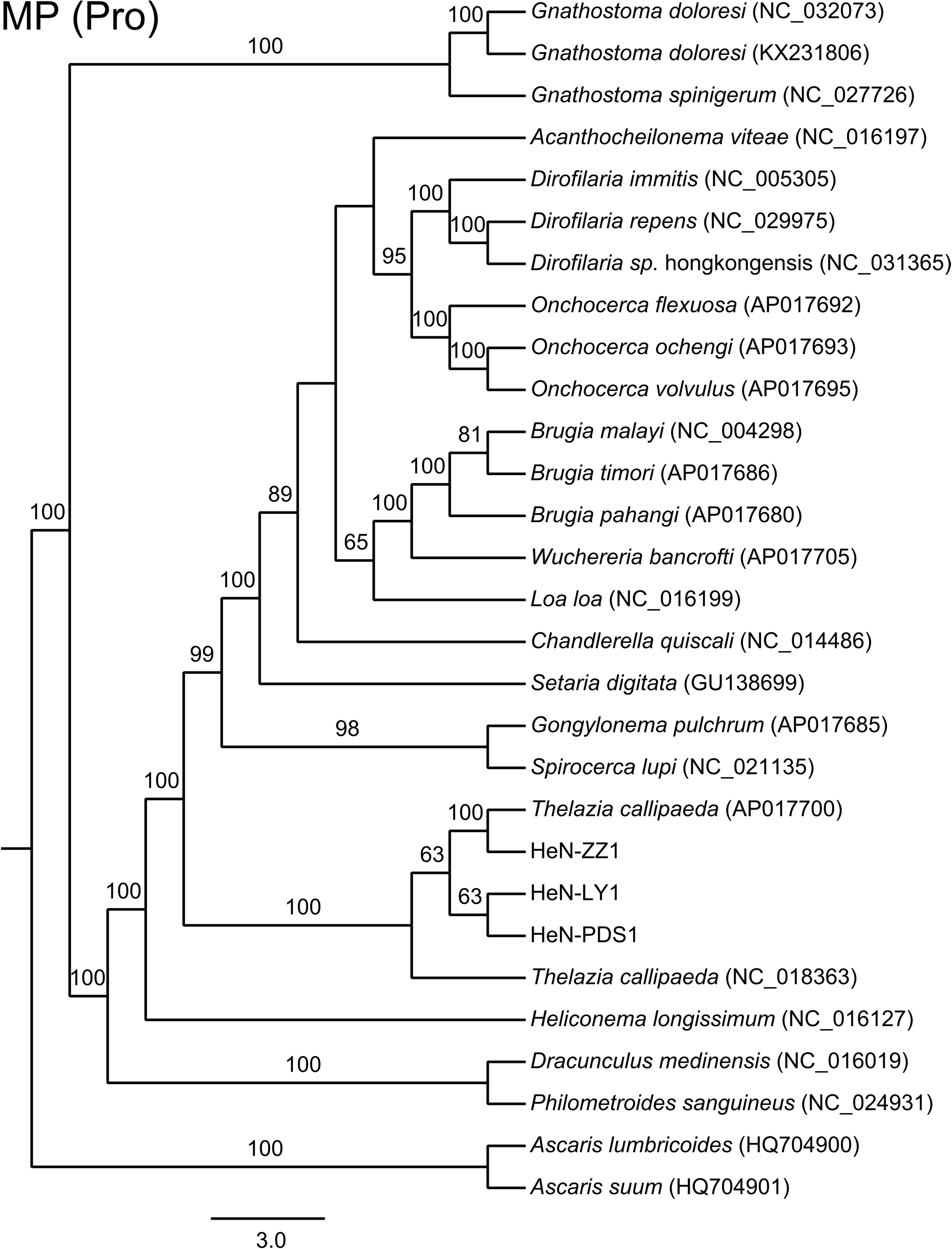


**Figure S2.** Maximum parsimony (MP) phylogenetic tree of collecting spirurid nematodes based on the analysis of PCGs.

**
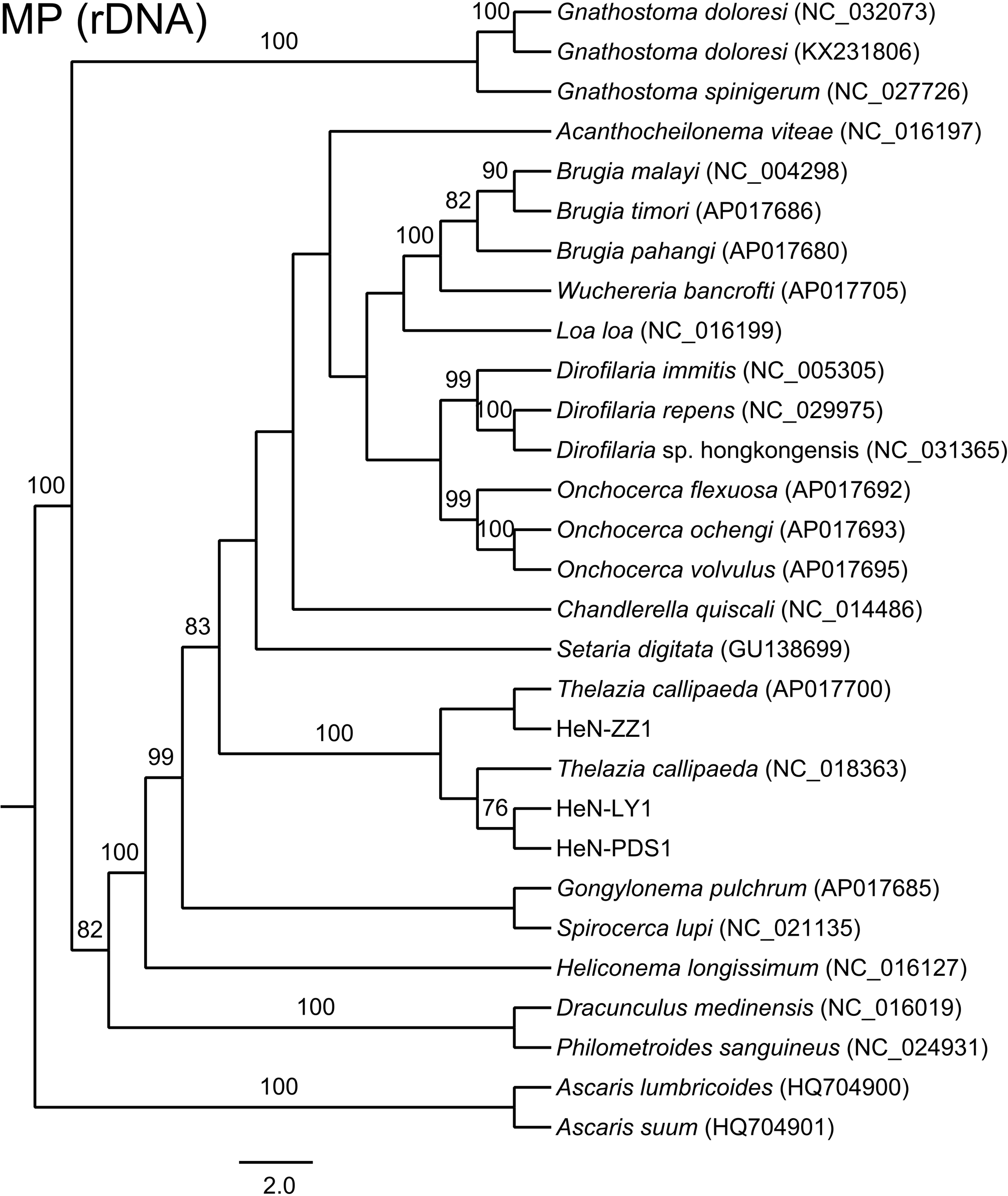
**

**Figure S3.** Maximum parsimony (MP) phylogenetic tree of collecting spirurid nematodes based on the analysis of 2 rRNA genes.


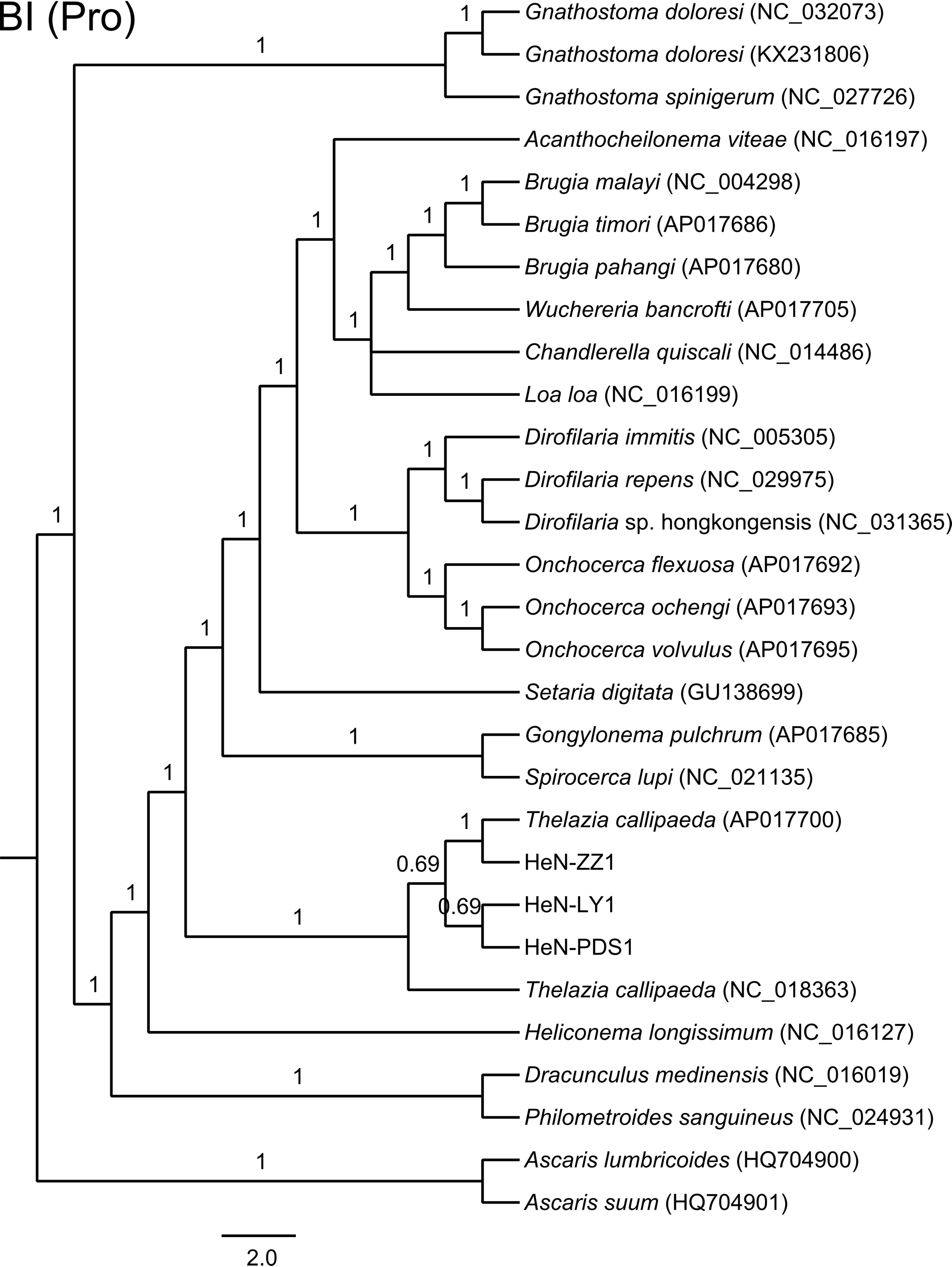


**Figure S4.** Bayesian phylogenetic tree of collecting spirurid nematodes based on the PCG analysis.

**
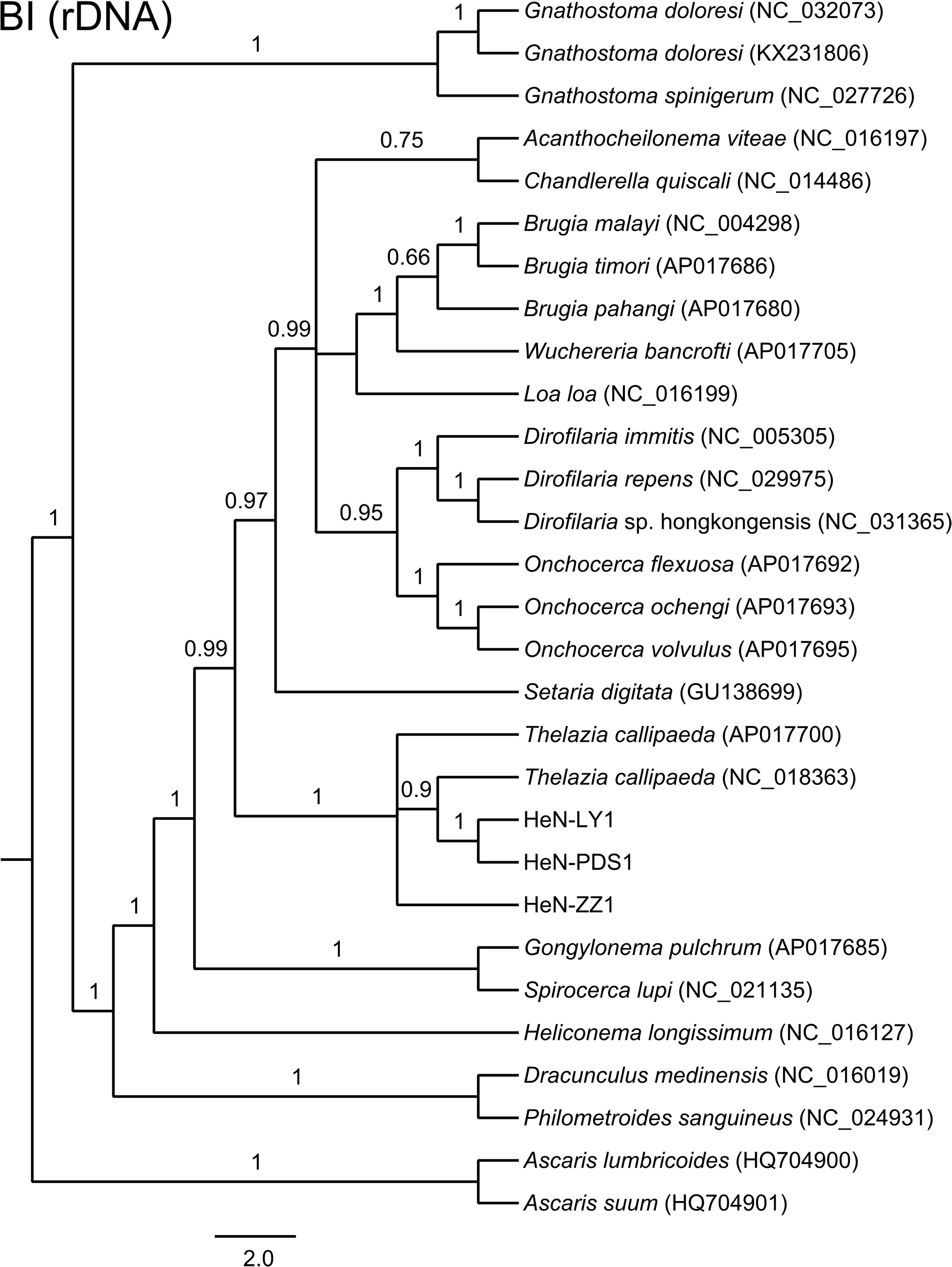
**

**Figure S5.** Bayesian phylogenetic tree of collecting spirurid nematodes based on the analysis of 2 rRNA genes.
